# Clustering of risk factors for cardiometabolic diseases in low-income, female adolescents

**DOI:** 10.1590/2359-3997000000083

**Published:** 2015-01-01

**Authors:** Elza M. F. S. de Melo, George D. Azevedo, João B. da Silva, Telma M. A. M. Lemos, Técia M. O. Maranhão, Ana K. M. S. O. Freitas, Maria H. Spyrides, Eduardo C. Costa

**Affiliations:** 1 Universidade Federal do Rio Grande do Norte Natal RN Brasil Programa de Pós-Graduação em Ciências da Saúde, Universidade Federal do Rio Grande do Norte (UFRN), Natal, RN, Brasil; 2 Departamento de Pediatria UFRN Natal RN Brasil Departamento de Pediatria, UFRN, Natal, RN, Brasil; 3 Departamento de Morfologia Centro de Biociências UFRN Natal RN Brasil Departamento de Morfologia, Centro de Biociências, UFRN, Natal, RN, Brasil; 4 Departamento de Educação Física Universidade do Estado do Rio Grande do Norte Mossoró RN Brasil Departamento de Educação Física, Universidade do Estado do Rio Grande do Norte (UERN), Mossoró, RN, Brasil; 5 Departamento de Análises Clínicas UFRN Natal RN Brasil Departamento de Análises Clínicas, UFRN, Natal, RN, Brasil; 6 Departamento de Obstetrícia e Ginecologia UFRN Natal RN Brasil Departamento de Obstetrícia e Ginecologia, UFRN, Natal, RN, Brasil; 7 Departamento de Estatística UFRN Natal RN Brasil Departamento de Estatística, UFRN, Natal, RN, Brasil

**Keywords:** Adolescents, risk factors, cardiovascular disease, obesity

## Abstract

**Objective:**

To assess the prevalence and clustering patterns of cardiometabolic risk factors among low-income, female adolescents.

**Materials and methods:**

Cross-sectional study involving 196 students of public schools (11-19 years old). The following risk factors were considered in the analysis: excess weight, central obesity, dyslipidemia, high blood pressure, and high fasting glucose. The ratio between observed and expected prevalence and its confidence interval were used to identify clustering of risk factors that exceeded expected prevalence in the population.

**Results:**

The most prevalent risk factors were dyslipidemia (70.9%), and central obesity (39.8%), followed by excess weight (29.6%), and high blood pressure (12.8%). A total of 42.9% of adolescents had two or more risk factors, and 24% had three or more. Excess weight, central obesity, and dyslipidemia were common risk factors in the clustering patterns that showed higher-than-expected prevalence.

**Conclusions:**

Clustering of risk factors (≥ two factors) among the adolescents showed considerable prevalence, and there was a non-casual coexistence of excess weight, central obesity, and dyslipidemia (mainly low HDL-cholesterol).

## INTRODUCTION

Biological (1,2) and behavioral (3,4) risk factors are associated with the development of cardiometabolic diseases. Although isolated risk factors have a specific impact on health, they are more frequently clustered in individuals (5). The clustering pattern of these risk factors have been investigated, including in adolescents (6-10), and this kind of analysis may subsidize the identification of risk factor clustering phenotypes in given populations (5,11), making clinical management easier by the use of specific preventive or therapeutic approaches.

It is important to emphasize that the atherosclerotic process may begin in childhood, and its severity is related with the number of simultaneous risk factors (7,12). Therefore, early morbimortality is related with the number of risk factors and their magnitude (13). Besides, there seems to be a synergistic, not simply an additive, relationship between risk factors (5,13). In Brazil, the prevalence of risk factors among female adolescents have increased significantly in the last decades. According to the Familiar Budget Research (POF) (14), the prevalence of overweight and obesity in 10 to 19-year-olds in 2008-2009 was 19.4 in the population, and 4.0% among females. In 1974-1975, according to the Nacional Study on Family Expenses (ENDEF) (15), these indices were 7.6 and 0.7%, a difference that demonstrates an important growth in excess weight in this population.

Although the last decades showed an increase in the number of studies on the prevalence of cardiometabolic risk factors (6-10), little is known about the simultaneous occurrence of these factors, or about their clustering pattern, especially in females. Therefore, the investigation of these aspects in female adolescents, specially in low-income populations that are seek attention at the Sistema Único de Saúde (SUS), may contribute to specific preventive and/or therapeutic strategies. Thus, the objective of the present study was to analyze the prevalence and clustering pattern of risk factors for cardiometabolic diseases in low-income, female adolescents.

## MATERIALS AND METHODS

This is an observational, transversal study. Female students (between 11 and 19 years of age) from public schools of a low-income region at the east region of the city of Natal/RN were analyzed. The neighborhood that was studied had mean nominal monthly earnings of 0.78 minimum wages; 80% of the private households in the neighborhood have *per capita* nominal monthly earnings of up to one minimum wage (Natal-RN: 45%) (16). There are 50,195 inhabitants in this neighborhood, from which 10,754 are between 10 and 19 years old. From these, approximately 50% (5,377) are females. In the present study, 196 pubescent adolescent students between 11 and 19 years of age that did not make use hormonal contraceptives were analyzed.

Data were collected from two Family Health Units between August 2010 and October 2012, after an invitation to take part in the study was sent to the parents or guardians of the adolescents of the two major public health schools (a municipal and a state school) in this neighborhood. Before that, the study was approved by the Ethics Committee of Universidade Federal do Rio Grande do Norte (UFRN; protocol no. 402/09). Parents or guardians of all volunteers signed an informed consent form before enrolling in the study, according to Resolution 196/96 from the Brazilian National Health Council.

The following inclusion criteria were used: i) age between 11 and 19 years old; ii) menarche having occurred at least one year before; iii) no use of hormonal contraceptives; iv) no continuous use of any medication; v) not being pregnant; vi) no physical problems that prevented anthropometric measurements; vii) *per capita* mean earnings of up to half minimum wage or family monthly earning of up to three minimum wages.

A multi-professional team that included physicians, a physical educator, a laboratory technician and a pharmacist-biochemist was responsible for the evaluations. Evaluations were carried out in the following order: i) anamnesis and clinical evaluation; ii) anthropometric measurements; iii) blood collection. The procedures are detailed below.

### Anamnesis and clinical evaluation

In this stage, the adolescents answered questions, in the presence of their parents or guardians, about their life habits, eating habits, previous and current diseases, vaccination and familiar disease background. In the clinical examination, timing of puberty (Tanner staging) was determined; thyroid evaluation, analysis of the presence of edema in lower limbs, cardiac and lung auscultation, and superficial and deep palpation of the abdomen were carried out. Blood pressure and cardiac rate at rest were also assessed. Blood pressure was carried out according to the VI Brazilian Guidelines on Hypertension (17), using an Onrom^®^ HEM-742 blood pressure monitor (oscillatory measurement) that was previously validated in Brazilian adolescents (18), with a cuff of adequate size.

### Anthropometric measurements

The following anthropometric measurements were carried out: body mass (kg), height (m) and waist circumference (cm). Body mass index (BMI, kg/m^2^) was calculated as the ratio between body mass (kg) and height squared (m^2^). For BMI stratification, the cutoff points proposed by the World Health Organization were used (19): “low weight” (Z score < -2), “eutrophic” (Z score > -2 and < +1), “overweigh” (Z score > +1 and < +2) and “obese” (Z score > +2). Waist circumference was measured in the mid-point between the iliac crest and the lowest rib at the end of a normal expiration, with a flexible but inelastic measuring tape (Sanny^®^, Brazil).

### Blood collection

Samples of venous blood (8 mL) were collected between 8 am and 10 am after a 12-hour fast. Serum glucose was measured using the glucose oxidase method. Total cholesterol, HDL-cholesterol and triglycerides were determined by a colorimetric assay (BioSystems^®^, Barcelona, Spain). LDL-cholesterol was calculated using the Friedewald equation (total cholesterol – [HDL-cholesterol + triglycerides/5]). All analyses were carried out by the Laboratório Integrado de Análises Clínicas (LIAC) at the Pharmacy School at Universidade Federal do Rio Grande do Norte.

### Risk factors and cutoff points

The following risk factors were considered in the analysis: i) excess weight: “overweight” or “obese” classification according to the World Health Organization (19); ii) central obesity: waist circumference ≥ 90^th^ percentile for ages between 10 and 16 years old and ≥ 80 cm above 16 years of age, according to the International Diabetes Federation (20); iii) dyslipidemia: total cholesterol ≥ 170 mg/dL, or LDL-cholesterol ≥ 130 mg/dL, or HDL-cholesterol < 45 mg/dL, or triglycerides ≥ 130 mg/dL, according to the V Brazilian Guidelines on Dyslipidemias and Atherosclerosis Prevention (21) for children and adolescents; iv) high blood pressure: systolic blood pressure ≥ 130 mmHg and/or diastolic blood pressure ≥ 85 mmHg (20); v) altered fasting glucose: blood glucose ≥ 100 mg/dL (20).

### Statistical analysis

The sample was characterized using means ± standard deviation. The initial analysis included the description of prevalence and the respective confidence intervals (CI95%) for the risk factors, either isolated or in combination. For the analysis of the clustering pattern of the risk factors, the ratio between observed and expected prevalence (O/E) for each of the possible combinations was calculated. Expected prevalence of an specific risk factor clustering pattern was calculated based on the individual probability of each risk factor according to its occurrence in the sample. For example, expected prevalence of the clustering between central obesity (OC), high BP, dyslipidemia (DISL), absence of altered blood glucose (GL) and excess weight (EP) was calculated as follows: *p*OC x *p*PA x *p*DISL x (1 – *p*GL) x (1 – *p*EP), where *p* is the probability (prevalence/100) of the factor in the studied sample (5,22). Therefore, it was possible to investigate which combinations were higher or lower than expected values, assuming that risk factors occur independently in the studied population. A statistically significant ratio is one that does not include the null value in its confidence interval. All analyzes were carried out in Stata 9.0 (STATA Corp., USA), at a 5% significance level.

## RESULTS

Table 1 shows the characteristics of the sample. Except for mean HDL-cholesterol, which was below the recommended value (≥ 45 mg/dL), all other biochemical, hemodynamic, and anthropometric variables were inside the normal range.

**Table 1:** Characteristics of the studied sample (n = 196)

Variables	Mean	Standard deviation
Age (years)	14.9	1.8
Age at menarche (years)	11.7	1.2
Body mass (kg)	54.3	11.2
Height (m)	1.57	0.06
Body mass index (kg/m^2^)	22.0	4.0
Waist circumference (cm)	78.6	9.4
Fasting glucose (mg/dL)	72.1	8.1
Total cholesterol (mg/dL)	134.2	24.6
HDL-cholesterol (mg/dL)	42.4	9.4
LDL-cholesterol (mg/dL)	77.4	23.2
Triglycerides (mg/dL)	72.1	29.0
Blood pressure (mmHg)		
Systolic blood pressure	114.8	11.8
Diastolic blood pressure	67.7	8.0

As for the economic status of the household, 166 adolescents (84.5%) presented household income between one to three minimum wages, and 30 (15.5%) of them, less than one minimum wage. In relation to parental education level, the father and/or mother of 158 (80.5%) adolescents completed primary education, and 34 (17.5%) had parents with complete or incomplete secondary education.

Table 2 shows the prevalence for the cardiometabolic risk factors analyzed in the present study. The most prevalent factors were dyslipidemia (70.9%) and central obesity (39.8%), followed by excess weight (29.6%), and high blood pressure (12.8%). None of the adolescents showed high fasting glucose. Specifically in relation to the dyslipidemia pattern, 67.3% (n = 132) presented low HDL-cholesterol, 4.1% (n = 8) showed hypertriglyceridemia, 1.5% (n = 3) had hypercholesterolemia, and 1.5% (n = 3) had high LDL-cholesterol. In relation to simultaneous risk factors, 42.9% of the adolescents showed two or more risk factors, and 24% showed three or more factors (Table 3).

**Table 2:** Prevalence of risk factors for cardiometabolic diseases in female adolescents in the study

Risk factors	n	% (CI95%)
Excess weight	58	29.6 (23.2-36.0)
Central obesity	78	39.8 (32.9-46.6)
Dyslipidemia	139	70.9 (64.5-77.3)
High blood pressure	25	12.8 (8.1-17.5)

Excess weight (18) = overweight or obesity, based on the body mass index; central obesity (19) = waist circumference ≥ 90^th^ percentile (10-16 years old) or ≥ 80 cm (≥ 16 years old); high fasting glucose (19) = ≥ 100 mg/dL; Dyslipidemia (20) = at least one altered result for the following markers: total cholesterol (≥ 170 mg/dL), LDL-cholesterol (≥ 130 mg/dL), HDL-cholesterol (< 45 mg/dL) or triglycerides (≥ 130 mg/dL); high blood pressure (19) = systolic blood pressure ≥ 130 mmHg and/or diastolic blood pressure ≥ 85 mmHg; CI95% = 95% confidnce interval.

**Table 3:** Clustering of risk factors for cardiometabolic diseases in female adolescents in the study

Risk factors	n	% (CI95%)
0	36	18.4 (13.0-23.8)
1	76	38.8 (32.0-45.6)
2	37	18.9 (13.4-24.4)
3	38	19.4 (13.9-24.9)
4	09	4.6 (1.7-7.5)
5	-	-

Table 4 shows observed and expected prevalence for each of the combinations of cardiometabolic risk factors analyzed. Two clustering patterns stood out: they include excess weight, central obesity, dyslipidemia, and high blood pressure (pattern 1); and excess weight, central obesity, and dyslipidemia (pattern 2). These patterns were more prevalent than expected if factors were independent from each other (if random). The other clustering patterns did not show higher-than-expected prevalence.

**Table 4:** Clustering pattern of the risk factors for cardiometabolic diseases in female adolescents in the study

No. of risk factors	EW	CO	HFG	DISL	HBP	n	O	E	O/E (CI95%)
4	+	+	-	+	+	09	4.6	1.1	4.3 (2.8-5.8)*
3	+	+	-	+	-	33	16.8	7.3	2.3 (1.7-2.9)*
3	+	+	-	-	+	01	0.5	0.4	1.1 (-1.7-4.0)
3	-	+	-	+	+	04	2.0	2.5	0.8 (-0.5-2.1)
2	+	+	-	-	-	09	4.6	3.0	1.5 (0.5-2.6)
2	+	-	-	+	-	32	16.3	11.0	1.5 (0.5-1.4)
2	-	+	-	+	-	15	7.7	17.3	0.4 (-0.1-1.0)
2	-	+	-	-	+	01	0.5	1.0	0.5 (-1.9-2.9)
2	-	-	-	+	+	07	3.6	3.8	0.9 (-0.1-1.9)

+ = presence of the risk factor; - = absence of the risk factor; EW = excess weight; CO = central obesity; HFG = high fasting glucose; DISL = dyslipidemia; HBP = high blood pressure; O = observed prevalence (%); E = expected prevalence (%); O/E = ratio between observed and expected prevalence; CI95% = 95% confidence interval; * = combinations that showed higher-than-expected ratios if the occurrence of the risk factors was independent.

## DISCUSSION

The objective of the present study was to analyze the prevalence and clustering pattern of risk factors for cardiometabolic diseases in low-income, female adolescents. The main findings were: i) dyslipidemia and central obesity were the most prevalent risk factors, followed by excess weight and high blood pressure; ii) about 25% of the adolescents analyzed presented three or four simultaneous cardiometabolic risk factors; iii) two clustering patterns (from nine that were observed) for the cardiometabolic risk factors were above expected prevalence, with excess weight, central obesity, and dyslipidemia as the most common factors.

Although knowledge about simultaneous risk factors is important, analyzing how these factors are combined seems more interesting, given the fact that knowledge on the existence of more prevalent phenotypes may support more specific preventive or therapeutic actions (23). From the 26 possible clustering patterns that included at least two of the risk factors analyzed, nine were observed in this study, and two showed prevalence higher than expected if risk factors were independent from each other (if they were random, see Table 4). This finding reinforces the idea that risk factors may occur simultaneously and in a dependent manner, increasing the risk of cardiovascular and/or metabolic disease. It is important to emphasize that this clustering pattern may be established early in life, as occurred in the adolescents analyzed in this study.

Previous studies demonstrated that clustering of cardiometabolic risk factors may occur in children, as well as in adolescents (6,7,9). It is important to emphasize the association between excess weight and the presence of clusters of cardiometabolic risk factors. Camhi and Katzmarzyk (9), analyzing data from the National Health and Nutrition Examination Survey (NHNES; 2,457 adolescents between 12-18 years old), observed a relationship between excess weight and clustering of risk factors. Using the BMI as a categorical variable, the authors demonstrated that 9%, 21% and 35% normal weight, overweight and obese adolescents, respectively, show clustering of two or more risk factors.

The presence of a cluster of risk factors may be an important risk predictor from the clinical practice standpoint. Shah and cols. (8) showed that adolescents and young adults (between 11-23 years old) with at least two simultaneous cardiovascular risk factors present early indicators of atherosclerosis, such as thicker and more rigid vessels, when compared with groups with less than two risk factors. The authors point out to the importance of early screening of cardiovascular risk factors in clinical practice in order to set up interventions in individuals at high risk for the development of atherosclerotic disease, that is, those that show two or more simultaneous risk factors. Considering this finding, it is important to emphasize that 42.9% of the adolescents evaluated in the present study showed the prevalence of two or more factors. Therefore, even though previous studies (23-26) have evidenced that the prevalence of overweight and obesity, which may seem to the trigger for several cardiovascular and metabolic changes, is greater in private schools and in better socioeconomic conditions, data of the present study show that low-income female adolescents also have an important cardiometabolic risk profile.

From the standpoint of the biological risk factors clustering phenotype in adolescents, the identification of common patterns at this age may aid clinical decision-making (therapy and prevention). Hong and cols. (6) evaluated the prevalence of metabolic syndrome and the clustering of risk factors in Vietnam adolescents (between 13-16 years old). These authors observed that overweight and obese adolescents showed greater prevalence of metabolic syndrome, no matter the diagnostic criterion. Similarly, in our study, the most common risk factor was plasma lipids changes (triglycerides and HDL-cholesterol), and the least prevalent one was altered fasting glucose. Main component analysis showed that obesity, hypertension, dyslipidemia, and hyperglycemia in females are responsible for 73.6% of the variance observed in metabolic syndrome.

In a multicentric study carried out in Iran (CASPIAN Study; n = 4,811) on the clustering of risk factors related to metabolic syndrome in children and adolescents (6-18 years old), Kelishadi and cols. (7) demonstrated that three risk factors were found in all age groups, no matter the sex, and were responsible for 87.4-90.8% of the metabolic syndrome variance in the population studied. They were: dyslipidemia, adiposity, and high blood pressure. Besides, the authors observed that waist circumference was a central and common factor in dyslipidemia and excess weight, reinforcing the role of central obesity in cardiometabolic changes. Once more, the clustering phenotype involving these three factors (obesity, dyslipidemia, and high blood pressure) was observed, as well as in the study by Hong and cols. (6). Similarly, we observed higher-than-expected prevalence for the phenotype involving excess weight, central obesity, dyslipidemia, and high blood pressure, which points out to a non-causal coexistence of these risk factors, as suggested by previous studies (6,7).

Aizawa and cols. (5) observed, in a large sample of Japanese patients over 40 years of age (41,819 men and 77,593 women), that almost all clustering of risk factors that had at least three of the five components used in the diagnosis of metabolic syndrome occurred above expected levels, indicating a non-causal clustering of risk factors. These authors reinforced the fundamental role that central obesity seems to have in integrating the different cardiometabolic changes and, as a consequence, in the clustering of these factors. Our data are in agreement with this hypothesis, as central obesity was a common factor in the two clustering patterns that occurred in higher-than-expected rates in the adolescents of the present study.

It is important to emphasize that the present study analyzed only adolescents that did not make use of hormonal contraceptives. From the clinical standpoint, there is evidence that the use of hormonal contraceptives leads to gain of body mass, although the definitive relationship between these factors has not been completely elucidated (27). In this sense, it is plausible to infer that low-income adolescents that make use of hormonal contraceptives may show even greater prevalence of cardiometabolic risk factors, as well as clustering of these factors.

Recently, da Silva and cols. (10), by means of transversal study in schools (n = 1,675; between 11-17 years old), evidenced clustering phenotypes related to behavioral and biological risk factors. In females, it was observed that the clustering phenotype involving waist circumference, high blood pressure, and low cardiorespiratory aptitude (biological factors) show higher-than-expected prevalence. In relation to behavioral factors, clustering phenotypes in which higher-than-expected prevalence was observed have smoking and alcohol consumption as common aspects. Besides, considering biological and behavioral risk factors, the authors demonstrated that 62% of the adolescents (male and females) have at least two risk factors for chronic non-transmissible diseases. These findings point out to the importance not limiting clinical screening of risk factors to biological factors alone, and to include behavioral factors, as well.

In summary, the most prevalent risk factors in low-income adolescents analyzed were dyslipidemia, central obesity, excess weight, and high blood pressure. About 40% of them presented two or more risk factors, and about 25% of them, three or four factors. In this population, the risk factor clustering phenotypes with higher-than-expected prevalence included excess weight, central obesity and dyslipidemia. From the clinical practice standpoint, it is important to consider risk factor clustering phenotypes in order to prevent early incidence in adults life of cardiometabolic diseases, such as systemic hypertension and type 2 diabetes. Besides, there is a need for investing in educational actions involving parents, adolescents, and schools on the importance of healthy habits, such as physical activity, adequate nutrition, and less sedentary behavior.

## References

[1] . Huang Y, Wang S, Cai X, Mai W, Hu Y, Tang H, et al. Pre hypertension and incidence of cardiovascular disease: a meta-analysis. BMC Med. 2013;11:177. 10.1186/1741-7015-11-177 PMC3750349 23915102

[2] . Anand SS, Dagenais GR, Mohan V, Diaz R, Probstfield J, Freeman R, et al. Glucose levels are associated with cardiovascular disease and death in an international cohort of normal glycaemic and dysglycaemic men and women: the EpiDREAM cohort study. Eur J PrevCardiol. 2012;19(4):755-64. 10.1177/1741826711409327 21551215

[3] . Lipton R, Cunradi C, Chen MJ. Smoking and all-cause mortality among a cohort of urban transit operators. J Urban Health. 2008;85(5):759-65. 10.1007/s11524-008-9295-6 PMC2527437 18553222

[4] . Lollgen H, Bockenhoff A, Knapp G. Physical activity and all-cause mortality: an updated meta-analysis with different intensity categories. Int J Sports Med. 2009;30(3):213-24. 10.1055/s-0028-1128150 19199202

[5] . Aizawa Y, Kamimura N, Watanabe H, Aizawa Y, Makiyama Y, Usuda Y, et al. Cardiovascular risk factors are really linked in the metabolic syndrome: This phenomenon suggests clustering rather than coincidence. Int J Cardiol. 2006;109(2):213-8. 10.1016/j.ijcard.2005.06.007 16023231

[6] . Hong TK, Trang NH, Dibley MJ. Prevalence of metabolic syndrome and factor analysis of cardiovascular risk clustering among adolescents in Ho Chi Minh City, Vietnam. Prev Med. 2012;55(5):409-11. 10.1016/j.ypmed.2012.09.002 22975412

[7] . Kelishadi R, Ardalan G, Adeli K, Motaghian M, Majdzadeh R, Mahmood-Arabi MS, et al. Factor analysis of cardiovascular risk clustering in pediatric metabolic syndrome: CASPIAN study. Ann Nutr Metab. 2007;51(3):208-15. 10.1159/000104139 17587791

[8] . Shah AS, Dolan LM, Gao Z, Kimball TR, Urbina EM. Clustering of risk factors: a simple method of detecting cardiovascular disease in youth. Pediatrics. 2011;127(2):e312-8. 10.1542/peds.2010-1125 PMC3025419 21242216

[9] . Camhi SM, Katzmarzyk PT. Prevalence of cardiometabolic risk factor clustering and body mass index in adolescents. J Pediatr. 2011;159(2):303-7. 10.1016/j.jpeds.2011.01.059 21429506

[10] . da Silva KS, Lopes AS, Vasques DG, da Costa FF, da Silva RCR. Simultaneidade dos fatores de risco para doenças crônicas não transmissíveis em adolescentes: prevalência e fatores associados. Rev Paul Pediatr. 2012;30(3):338-45.

[11] . Wilson PWF, Kannel WB, Silbershatz H, D’Agostino RB. Clustering of metabolic factors and coronary heart disease. Arch Intern Med. 1999;159(10):1104-9. 10.1001/archinte.159.10.1104 10335688

[12] . Sociedade Brasileira de Cardiologia, Departamento de Aterosclerose. I diretriz de prevenção da aterosclerose na infância e na adolescência. Arq Bras Cardiol. 2005;85:3-36. 16597097

[13] . Rabelo LM. Fatores de risco para doença aterosclerótica na infância. J Pediatr. 2001;77(Supl. 2):s153-64. 10.2223/jped.303 14676878

[14] . Instituto Brasileiro de Geografia e Estatística (IBGE). Pesquisa de Orçamentos Familiares 2008-2009: antropometria e estado nutricional de crianças, adolescentes e adultos no Brasil. Rio de Janeiro: IBGE, 2010.

[15] . Instituto Brasileiro de Geografia e Estatística (IBGE). Estudo Nacional de Despesas Familiares: 1974-1975. Rio de Janeiro: IBGE, 1977.

[16] . Instituto Brasileiro de Geografia e Estatística (IBGE). Censo Demográfico 2010. Rio de Janeiro: IBGE, 2010. Available from: <http://www.cidades.ibge.gov.br/xtras/perfil.php?lang=&codmun=240810&search=rio-grande-do-norte|natal>. Accessed on: Jun 3, 2015.

[17] . Sociedade Brasileira de Cardiologia; Sociedade Brasileira de Hipertensão; Sociedade Brasileira de Nefrologia. VI Brazilian Guidelines on Hypertension. Arq Bras Cardiol. 2010;95(1 Suppl):1-51. 21085756

[18] . Christofaro DG, Fernandes RA, Gerage AM, Alves MJ, Polito MD, Oliveira AR. Validation of the Omron HEM 742 blood pressure monitoring device in adolescents. Arq Bras Cardiol. 2009;92(1):10-5. 10.1590/s0066-782x2009000100003 19219259

[19] . World Health Organization. Child growth standards based on length/height, weight and age. Acta Paediatr Suppl. 2006;450:76-85. 10.1111/j.1651-2227.2006.tb02378.x 16817681

[20] . Zimmet P, Alberti KG, Kaufman F, Tajima N, Silink M, Arslanian S, et al. The metabolic syndrome in children and adolescents – an IDF consensus report. Pediatr Diabetes. 2007;8:299-306. 10.1111/j.1399-5448.2007.00271.x 17850473

[21] . Xavier HT, Izar MC, Faria Neto JR, Assad MH, Rocha VZ, Sposito AC, et al. V Diretriz Brasileira de Dislipidemias e Prevenção da Aterosclerose. Arq Bras Cardiol. 2013;101(4 Suppl. 1):1-20. 10.5935/abc.2013S010 24217493

[22] . Costa FF, Montenegro VB, Lopes TJ, Costa EC. Combination of risk factors for metabolic syndrome in the military personnel of the Brazilian Navy. Arq Bras Cardiol. 2011;97(6):485-92. 10.1590/s0066-782x2011005000113 22030564

[23] . Balaban G, Silva GAP, Motta MEFA. Prevalência de sobrepeso e obesidade em escolares de diferentes classes socioeconômicas em Recife, PE. Pediatria. 2001;23(4):285-9.

[24] . Silva GAP, Balaban G, Motta MEFA. Prevalência de sobrepeso e obesidade em crianças e adolescentes de diferentes condições socioeconômicas. Rev Bras Saúde Matern Infant. 2005;5(1):53-9.

[25] . da Costa RF, Cintra IP, Fisberg M. Prevalência de sobrepeso e obesidade em escolares da cidade de Santos, SP. Arq Bras Endocrinol Metab. 2006;50(1):60-7. 10.1590/s0004-27302006000100009 16628276

[26] . Ricardo GD, Caldeira GV, Corso ACT. Prevalência de sobrepeso e obesidade e indicadores de adiposidade central em escolares de Santa Catarina, Brasil. Rev Bras Epidemiol. 2009;12(3):424-35.

[27] . Gallo MF, Lopez LM, Grimes DA, Carayon F, Schulz KF, Helmerhorst FM. Combination contraceptives: effectson weight. Cochrane Database Syst Rev. 2014;1:CD003987. 10.1002/14651858.CD003987.pub5 PMC10640873 24477630

